# Pediatric patients at risk for fever in chemotherapy-induced neutropenia in Bern, Switzerland, 1993-2012

**DOI:** 10.1038/sdata.2018.38

**Published:** 2018-03-13

**Authors:** Annina N. von Allmen, Maxime G. Zermatten, Kurt Leibundgut, Philipp Agyeman, Roland A. Ammann

**Affiliations:** 1 Department of Pediatrics, Inselspital, Bern University Hospital, University of Bern, CH-3010 Bern, Switzerland

**Keywords:** Paediatric cancer, Bacterial infection, Fever

## Abstract

Fever in neutropenia (FN) is the most frequent potentially life threatening complication of chemotherapy for cancer. Prediction of the risk to develop FN during chemotherapy would allow for targeted prophylaxis. This retrospective, single centre cohort study in pediatric patients diagnosed with cancer before 17 years covered two decades, 1993 to 2012. The 583 (73%) of 800 patients diagnosed with cancer who had received chemotherapy were studied here. Data on 2113 observation periods was collected, defined by stable combinations of 11 predefined characteristics potentially associated with FN. They covered 692 years of cumulative chemotherapy exposure time, during which 712 FN episodes were diagnosed, 154 (22%) of them with bacteremia. The risk to develop FN and FN with bacteremia remained stable over time. These data can mainly be used to study FN risks over time and between centers, and to derive or externally validate FN risk prediction rules.

## Background and Summary

Fever in neutropenia (FN) is the most frequent potentially life-threatening complication of chemotherapy in children and adolescents with cancer^1^. About half of the children treated with chemotherapy for cancer develop at least one FN episode^2,3^. To reduce the risk to develop FN during chemotherapy, non-pharmacological measures, prophylactic antibiotics, and granulocyte colony-stimulating factor can be applied prophylactically^4,5^. The current standard therapy for FN includes emergency hospitalisation and empirical administration of intravenous broad-spectrum antibiotics^6^. This approach reduced FN mortality from around 30% in the 1970s to around 1% in the late 1990s (ref. 7).

Both prophylactic and therapeutic FN directed measures generate relevant costs, can stimulate bacterial resistance and can lead to decreased quality of life for patients and their families^5,6,8^. Efficient risk prediction might reduce these drawbacks. Considerable evidence has been published on the prediction of different outcomes in patients diagnosed with FN. Correspondingly, risk adapted FN therapy based on validated risk prediction rules is currently finding its way into routine management in pediatric oncology^6,9–14^. In contrast, there is only scarce and non-validated evidence how to predict the risk to develop FN during chemotherapy^15^. Prophylaxis is usually restricted to patients with acute myeloid leukemia^5,16^. Risk-adapted and thus targeted FN prophylaxis is far from clinical routine in the majority of patients^15^.

This study in children and adolescents treated with chemotherapy for cancer aimed to collect long-term data on 11 predefined patient- and treatment-related characteristics potentially associated with FN, on FN itself, and on the specifically relevant subgroup of FN with bacteremia. The main clinical motivation of this study was to generate data for the development of rules predicting the risk of FN during chemotherapy in pediatric patients with cancer, finally leading to evidence-based targeted prophylaxis in these patients.

This study was designed as a retrospective, single centre cohort study in pediatric patients diagnosed with cancer before 17 years, covering two decades, 1993 to 2012. All clinical information was directly extracted from patient charts.

**Figure 1:**
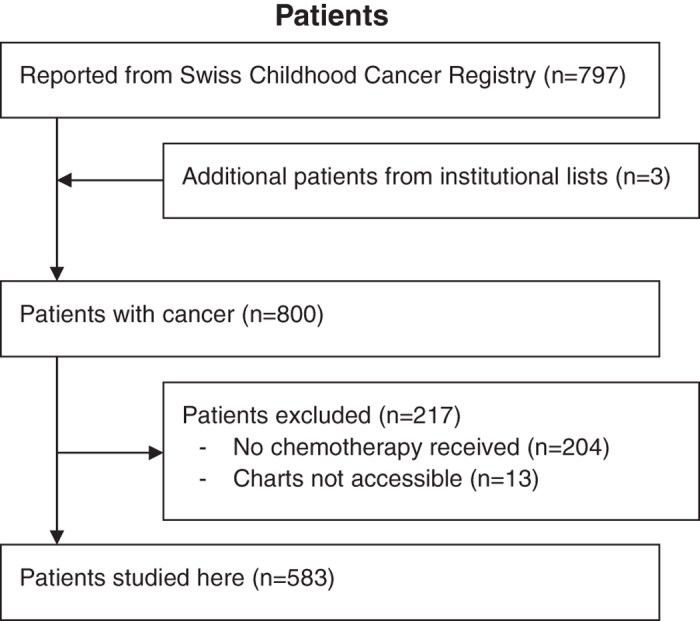
Patients flowsheet.

In total, 800 patients were found who had been diagnosed with cancer below the age of 17 years in Bern from 1993 to 2012. Of these, 596 (75%) were reported to have received chemotherapy. Charts were not accessible in 13 (2.2%). The remaining 583 (98%) patients were studied here (Figure 1). The slight preponderance of male patients (327, 56%), the distribution of age at diagnosis, and the distribution of diagnoses were all compatible with the known distributions of pediatric patients with cancer in developed countries.

A total of 2,113 observation periods, defined by a constant combination of the 11 characteristics, were found (median per patient, 3; range, 1 to 18; interquartile range (IQR), 2 to 5) (File data.17B.ObservationPeriods.csv. Data Citation 1). Their median duration was 64 days (range, 1 to 866; IQR, 25 to 157), with a cumulative duration of 252,724 days (692 years) chemotherapy exposure time (CET).

During the cumulative CET of 692 years, 846 FN episodes were known to be clinically diagnosed (rate, 1.22 per year; exact 95% confidence interval (CI), 1.14 to 1.31). Criteria of FN were not ascertainable in 43 (5%) of these episodes, and were not fulfilled in 91 (11%). The remaining 712 FN episodes (84%) were studied here (rate, 1.03 per year of CET; exact 95% CI, 0.95 to 1.11).

At least one FN episode was recorded in 294 (50%) of the 583 patients (median, 1; range, 0 to 9; IQR, 0 to 2). Bacteremia was detected in 154 (22%) of the 712 FN episodes in 111 (19%) patients. Over the time studied, the risk to develop FN (rate ratio per decade, 0.97; exact 95% CI 0.72 to 1.30; p=0.83, results of mixed Poisson regression) and FN with bacteremia (rate ratio per decade, 0.88; exact 95% CI, 0.38 to 2.04; p=0.76) did not change significantly.

These data can mainly be used (1) to study the risk to develop FN during chemotherapy over time and between centers; (2) to derive risk prediction rules for FN, and for FN with bacteremia; (3) to derive corresponding clinical decision rules for risk-adapted, targeted FN prophylaxis during chemotherapy; and (4) to externally validate corresponding rules derived from other datasets.

## Methods

### Study design

A retrospective, single site cohort study covering two decades, from 1993 to 2012, was performed at the Division of Pediatric Hematology and Oncology, Department of Pediatrics, Inselspital, Bern University Hospital, University of Bern, Switzerland.

This study was approved by the Institutional Review Board (Direktion Lehre und Forschung, Inselspital Bern; registration number, 13-06-11; last update, April 02, 2014), including waiver of informed consent. On June 30, 2014, data were fully anonymised before analysis, in order to comply with the requests of the new Swiss Federal Law on Human Research.

### Patients and their clinical management

A clinically useful extended cancer definition was used that included leukemia, lymphoma, malignant solid tumors, benign tumors of the central nervous system, and Langerhans cell histiocytosis^17^. All children and adolescents diagnosed with cancer and treated with chemotherapy were eligible. Age at diagnosis of malignancy and of FN episodes was restricted to ≤17 years. Patients were primarily identified via the Swiss Childhood Cancer Registry^17^. In order to reduce recruitment bias, information on patients was complemented using clinically used patients lists for the entire period, and patient lists from earlier research projects for a part of the period covered here^3,15,18–20^. Information on the characteristics of the patients studied, at start of study for the individual patients, is published (File data.17B.ObservationPeriods.csv. Data Citation 1).

Most patients were treated for their cancer according to established international protocols. Clinical management regarding prophylaxis and treatment of FN essentially remained unchanged during the entire study period: Patients did not receive any antibiotic prophylaxis beyond prophylaxis against *Pneumocystis jiroveci* pneumonia with oral trimethoprim / sulfamethoxazole, which was replaced by inhaled pentacarinate in selected patients^15^. Daily subcutaneous granulocyte colony-stimulating factor (G-CSF) was applied if requested by protocol^15^.

### Data collection

Information on chemotherapy, on FN episodes, on the detection of bacteremia in FN, and on 11 clinical characteristics potentially associated with FN was retrospectively extracted from patient charts as described^15^. Three of these 11 characteristics were patient related (gender, age at diagnosis, year of diagnosis), two were disease related (diagnostic group, relapse status), and six were related to therapy and course of disease (chemotherapy intensity, presence of central venous access device (CVAD), bone marrow involvement with ≥5% leukemic cells or any detectable malignant cells in solid tumors, time after diagnosis, prior episodes of FN, prior episodes of FN with bacteremia).

### Observation periods

The CET was defined as the cumulative duration of chemotherapy plus 3 weeks accounting for neutropenia developing after cessation of chemotherapy. The CET in a specific patient was split into a non-predefined number of observation periods. They were defined by a constant combination of the 11 characteristics potentially associated with the risk to develop FN described above. By definition, 8 of these 11 characteristics (2 disease related and 6 related to therapy and course of disease) can change over time. As an example, the induction therapy of a patient with newly diagnosed acute lymphoblastic leukemia is usually split into at least three observation periods: The first is characterized by the presence of bone marrow involvement, by definition, and the absence of a CVAD. The second starts on the day after insertion of a CVAD and is characterized by continued bone marrow involvement and the presence of a CVAD. The third starts on the day after diagnosis of remission and is characterized by the absence of bone marrow involvement with continued presence of a CVAD. Correspondingly, multiple observation periods were defined in the vast majority of patients. This allows for future analysis of associations of characteristics with FN, and FN with bacteremia. Information on the observation periods studied is published (File data.17B.ObservationPeriods.csv. Data Citation 1).

Given that for nearly all patients more than one observation period was recorded, that the length of these observation periods varies importantly, and that more than one FN episode can be recorded in one observation period, mixed Poisson regression seems to be an adequate type of analysis for these data, while time-to-event based analyses are not.

### Fever in neutropenia

An episode of FN was defined as fever in a patient with severe chemotherapy-induced neutropenia. Until July 8, 2007, fever was defined as an axillary temperature ≥38.5 °C persisting ≥2 h, or a single temperature ≥39.0 °C. Starting July 9, 2007, the temperature measurement method was changed from axillary to infrared tympanic thermometry, and the rule defining fever was simplified to a single tympanic temperature ≥39.0 °C^19^ for clinical reasons. In the setting of rising temperatures, the different limits used for the different measurement methods have been shown to be comparable^21^. Severe neutropenia was always defined as an absolute neutrophil count <0.5 G/L^22^. Multiple FN episodes per patient were allowed. All FN episodes fulfilling these criteria, even if fever occurred after interventions like transfusion of blood products or after cytarabine treatment, were included. However, episodes of FN additionally diagnosed for clinical reasons at lower temperatures and/or with an absolute neutrophil count ≥0.5G/L were not studied here^23^. Bacteremia was defined as the detection of any bacteria in any blood culture from the beginning until the end of the FN episode.

Emergency hospitalization and empirical broad-spectrum intravenous antimicrobial therapy, usually ceftriaxone plus amikacin, were routinely applied. Children were discharged and antibiotics were usually stopped if children were afebrile and well for 48 h, if there was no evidence for severe bacterial infection, and if there were any signs of bone marrow reconstitution^18^.

The beginning of an FN episode was defined as the time point when the FN criteria were fulfilled. The end of this episode was defined as the time point when antibiotics were stopped, the patient was discharged, or chemotherapy was restarted, whichever occurred earlier. If the FN criteria were fulfilled again after this time point, this counted as a further FN episode. Correspondingly, multiple FN episodes per observation period were possible. Taken together with the stopping criteria mentioned above, FN episodes could thus end despite continued neutropenia, and second FN episodes could be diagnosed within one episode of neutropenia.

### Data classification

Chemotherapy was classified into 4 levels of myelosuppressive intensity according to the expected duration of severe neutropenia as described^15,18^, which is an extension of an earlier model using only 2 levels^24^ (Table 1). This classification does not cover the additional risk of infection due to new therapeutic agents like rituximab that do not lead to relevant neutropenia.

In order to comply with the full anonymization mentioned above, age at diagnosis was split into 4 categories covering 4 years each (<4 years, 4 to 7.99 years, 8 to 11.99 years, and ≥12 years), and the time of observation was split into 5 periods of 4 years each (1993 to 1996, 1997 to 2000, 2001 to 2004, 2005 to 2008, and 2009 to 2012).

## Data Records

A single data records resulted from this study. It contains information on the 2,113 observation periods studied in these 583 patients (File data.17B.ObservationPeriods.csv. Data Citation 1) (Table 2).

Information on patients at time of study entry can be extracted from this file by selecting the first observation period per patient (LINE.PER.PAT=1).

Detailed information on variable specifications is included in a readme file (File data.17B.ReadMe.csv. Data Citation 1).

## Technical Validation

### Reduction of recruitment bias

Patients were primarily identified via the Swiss Childhood Cancer Registry^17^. In order to reduce recruitment bias, information on patients was complemented by clinically used institutional patients lists for the entire period, and patient lists from earlier research projects for a part of the period covered here^3,15,18–20^ (Figure 1).

### Increasing reliability of information on FN episodes

A simple restricted definition of FN episodes, based on verifiable quantitative information both on fever and on neutropenia was used. Correspondingly, FN episodes additionally diagnosed clinically when fever and or neutropenia limits had not been reached^23^, or when these limits were not ascertainable, were excluded.

A simple definition of bacteremia was used as well, without the need of partly subjective judgment if detection of a common commensal in a blood culture should be considered as infection, i.e., bacteremia, or contamination.

In case of inconsistencies or of unclear information found in the charts, an experienced pediatric haematologist-oncologist (RAA) consulted patient charts again and resolved these questions.

## Additional information

**How to cite this article**: von Allmen A. N. *et al.* Pediatric patients at risk for fever in chemotherapy-induced neutropenia in Bern, Switzerland, 1993-2012. *Sci. Data* 5:180038 doi: 10.1038/sdata.2018.38 (2018).

**Publisher’s note**: Springer Nature remains neutral with regard to jurisdictional claims in published maps and institutional affiliations.

## Supplementary Material



## Figures and Tables

**Table 1:** Myelosuppressive intensity of chemotherapy.

**Level**	**Intensity**	**Expected duration of severe neutropenia**
1	Minimally myelosuppressive	None expected
2	Briefly myelosuppressive	≤10 days
3	Strongly myelosuppressive	>10 days
4	Myeloablative	Hematopoietic stem cell recovery required
Modified from refs 15 and 18, plus ref. 23 regarding the distinction of level 2 versus 3.		

**Table 2:** Overview.

**Observations**	**Time covered**	**Center**	**Data source**	**Data**
Patients	1993 to 2012^a^	Bern^a^	Swiss Childhood Cancer Registry^17^Institutional patient lists	data.17B. ObservationPeriods.csv [LINE.PER.PAT=1]
Observation Periods	1993 to 2012^a^	Bern^a^	Chart records	data.17B.ObservationPeriods.csv

^a^Identical for both patients and observation periods.

## References

[d1] Figsharevon Allmen A. N. Zermatten M. G. Ammann R. A 2017 https://doi.org/10.6084/m9.figshare.4765216.v5

